# The Phenylpropanoid Case – It Is Transport That Matters

**DOI:** 10.3389/fpls.2018.01610

**Published:** 2018-11-01

**Authors:** Wanda Biała, Michał Jasiński

**Affiliations:** ^1^Department of Plant Molecular Physiology, Institute of Bioorganic Chemistry, Polish Academy of Sciences, Poznań, Poland; ^2^Department of Biochemistry and Biotechnology, Poznań University of Life Sciences, Poznań, Poland

**Keywords:** phenylpropanoids, transport, intermediates, metabolons, membrane transporters

## Abstract

Phenylpropanoids fulfill numerous physiological functions, essential for plant growth and development, as well as plant–environment interactions. Over the last few decades, many studies have shown that exquisite regulatory mechanisms at multiple levels control the phenylpropanoid metabolic pathway. Deciphering this pathway not only provides a greater, basic understanding of plant specialized metabolism, but also enhances our ability to rationally design plant metabolic pathways for future applications. Despite the identification of the participating enzymes of this complex, biosynthetic machinery, we still lack a complete picture of other genes, enzymes, and metabolites essential for regulation and compartmentation/distribution of phenylpropanoids. Compartmentation, as well as distribution, are critical for the fate/functioning of those molecules, and their effective biosynthesis. At the cellular level, we have narrowed down our understanding of these processes to organelles. Furthermore, various, overlapping, but not exclusive scenarios of phenylpropanoid distribution within the cell have also been described. The cross-membrane dynamics, but also intercellular communication of different branches from phenylpropanoid biosynthesis have become an exciting research frontier in plant science. The intra- and intercellular channeling of intermediates by various transport mechanisms and notably membrane transporters could be a meaningful tool that ensures, *inter alia*, efficient metabolite production.

## Introduction

The phenylpropanoid pathway is one of the most frequently investigated metabolic routes, among secondary metabolites. The existence of enzymatic reactions, where phenylalanine is converted to hydroxycinnamic acid, dates back to 450 million years ago (Fellenberg and Vogt, 2015), coinciding with colonization of the terrestrial environment by plants (Lanfranco et al., 2016). Products of the phenylpropanoid pathway are involved in many aspects of plant growth, structural support, and response to the stimuli inextricably associated with the life on land. Not only do they play a crucial role in stress response upon variation of light (Yang et al., 2018) and mineral shortage (Clemens and Weber, 2016), but they are also key mediators of the plant interactions with other organisms (Naoumkina et al., 2010; Shalaby and Horwitz, 2015; Liu and Murray, 2016). The utility of phenylpropanoids is a matter of being in the right place at the right time. This is tightly controlled, not only at the biosynthesis level, but also by various distribution systems. The latter comprise, *inter alia*, membrane transporters which participate in the circulation of both theintermediates and final products. This mini review is intended to position transport as a notable part of the regulatory network, tuning the phenylpropanoid biosynthesis according to the plant’s needs.

## Biosynthesis of Phenylpropanoids – a Dynamic Complexity

Phenylpropanoid metabolism generates an enormous array of secondary metabolites, based on the few intermediates of the shikimate pathway (Vogt, 2010). The shikimate pathway is a source of phenylalanine and the entry point leading to the biosynthesis of phenylpropanoids. The so-called central phenylpropanoid pathway is defined by three enzymatic activities: (i) the phenylalanine deamination by phenylalanine ammonia-lyase (PAL) to the *trans*-cinnamic acid, (ii) the *trans*-cinnamic acid hydroxylation to the 4-coumarate, as a resulting from cinnamic acid 4-hydroxylase (C4H) activity, and finally (iii) the 4-coumarate conversion to the 4-coumaroyl-CoA by 4-coumarate-CoA ligase (4CL). In many cases, genes from the central phenylpropanoid pathway are present in multiple copies. For instance, the *PAL* genes include six isoforms in Medicago (*Medicago truncatula*), five in poplar (*Populus trichocarpa*), nine in rice (*Oryza sativa*), and four in *Arabidopsis thaliana*. Various isoforms differ in terms of localization and activity. For instance, the *Arabidopsis PAL1*, *PAL2*, and *PAL4* are expressed at relatively high levels in stems during the later stages of development, with *PAL1* expression localized in the vascular tissue, and *PAL2* and *PAL4* both expressed in seeds. PAL1 and PAL2 were shown to be involved in flavonoid biosynthesis (Olsen et al., 2008), while the two others were suggested to participate in lignin formation (Huang et al., 2010). Similarly to *PAL* in *Arabidopsis*, *4CL* possesses four isoforms. The *4CL3* is expressed in a broad range of cell types, and is predominantly associated with flavonoid biosynthesis. *4CL1*, *4CL2*, and *4CL4* revealed co-expression with lignin biosynthetic genes (Ehlting et al., 1999; Li et al., 2015). The product of 4CL activity, the *p*-Coumaroyl-CoA is a crucial intermediate in the phenylpropanoid pathway. It is a precursor for: (i) monolignol, (ii) coumarin, (iii) stilbene, as well as (iv) (iso)flavonoid biosynthesis (Kutchan et al., 2015).

## Metabolons and Phenylpropanoid Pathway

**FIGURE 1:**
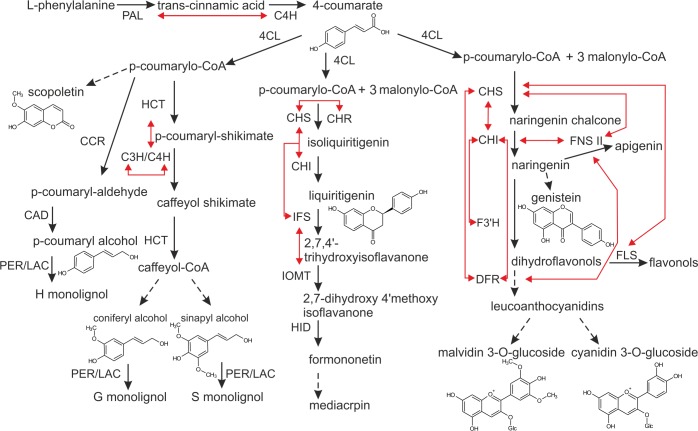
Scheme of the phenylpropanoid pathway. Direct interactions between particular biosynthetic enzymes, demonstrated by means of co-immunoprecipitation, BiFC or FRET, are illustrated by red arrows. Discontinuous lines indicate that certain steps along the pathway are not included in the figure. The enzymes are: PAL, phenylalanine ammonia-lyase; C4H, cinnamic acid 4-hydroxylase; 4CL, 4-coumarate:CoA ligase; CCR, cinnamoyl CoA reductase; CAD, cinnamoyl alcohol dehydrogenase; PER/LAC, peroxidase/laccase; HCT, shikimate hydroxycinnamoyl transferase; C3H/C4H, 4-coumarylshikimate 3-hydroxylase/cinnamate 4-hydroxylase; CHS, chalcone synthase; CHR, chalcone reductase; CHI, chalcone isomerase; IFS, isoflavone synthase; IOMT, isoflavone *O*-methyltransferase; HID, hydroxyisoflavanone dehydratase; F3H, flavanone 3 hydroxylase; DFR, dihydroflavonol 4-reductase; FNSII, flavanone synthase II; FLS, flavonol synthase.

The cooperating enzymes from the phenylpropanoid pathway were proposed to be organized into complexes called metabolons, and a number of reviews may serve as evidence in this respect (Winkel-Shirley, 1999; Jørgensen et al., 2005; Sweetlove and Fernie, 2013; Laursen et al., 2015; Bassard and Halkier, 2018). The term “metabolon” encompasses multienzymatic complexes bound to the cellular structural elements – membranes. Most metabolon models are based on a dynamic, non-covalent aggregation of components on the endoplasmic reticulum (ER) surface. Enzymes like chalcone synthase (CHS), chalcone reductase (CHR) are cytoplasmic enzymes however, other ones, like C4H or isoflavones synthase (IFS), are lodged in the ER, anchoring the biosynthetic enzyme complex (Dastmalchi et al., 2016). Organization of enzymes in metabolons is, at the cellular level, a way to optimize biosynthesis. It provides: (i) direct transport of intermediates between successive enzymes, hence increasing local concentration of the substrate around the enzyme active center, (ii) minimization of highly biologically active and potentially toxic intermediates within the cell, as well as (iii) coordination of reactions leading to different branches of pathways with shared enzymes or intermediates (Jørgensen et al., 2005; Bassard and Halkier, 2018). In the phenylpropanoid pathway, intracellular interactions between biosynthetic enzymes were shown for the central phenylpropanoid pathway – where PAL and C4H colocalize in the ER (Achnine et al., 2004), as well as for particular branches leading to the formation of (iso)flavonoids, monolignols, and anthocyanins (Figure 1). Key flavonoid enzymes exhibit multidirectional interactions: CHS-chalcone isomerase (CHI) (Saslowsky and Winkel-Shirley, 2001), CHS-CHR, IFS-upstreaming enzymes (Dastmalchi et al., 2016) and IFS- isoflavone *O*-methyltransferase (IOMT) (Liu and Dixon, 2001). In the anthocyanin route, flavonol synthase (FLS), and dihydroflavonol 4-reductase (DFR) were shown to interact with CHS in a competitive manner (Crosby et al., 2011), while flavanone 3-hydroxylase (F3′H) was indicated to interact with CHI (Burbulis and Winkel-Shirley, 1999). Additionally, flavone synthase II (FNS II) was shown to interact with DFR, as well as with the upstreaming enzymes like CHS and CHI, while the latter also interacts with CHS and DFR (Fujino et al., 2018). In the branch leading to lignin precursors, C4H and *p*-coumaroylshikimate 3′-hydroxylase (C3′H) were found to colocalize in the ER and being connected to shikimate hydroxycinnamoyl transferase (HCT) (Chen et al., 2011; Bassard et al., 2012). The existence of metabolons was also demonstrated in primary metabolism, for instance in fatty acid biosynthesis (Kwiatkowska et al., 2015), purine synthesis (Kyoung et al., 2015), or Krebs cycle (Wu et al., 2015). These systems involve stable enzyme associations that are suitable for experimental analysis. Potential metabolons formed in metabolic channeling of secondary metabolites in plants seem to be more dynamic and transient, according to the required plasticity in response to wide spectrum of stimuli, thus their existence is much more difficult to investigate and demonstrate.

## Intercellular Metabolons Cooperation?

When the concept of metabolons was conjectured the main feature of the model was the assumption that all stages of biosynthesis/intermediates sharing occur in a single cell. Recently, transient and dynamic metabolons formation/cooperation has been proposed as means of swift adaptation of the metabolite profile to environmental changes (Bassard and Halkier, 2018). Interestingly, in the opium poppy (*Papaver somniferum*), the spatial distribution of alkaloid biosynthesis and the cell-specific localization of key enzymes, which represent different pathway branches of the alkaloid, have been demonstrated. Such a separation of the intermediates in different cell types might help to avoid undesired secondary modifications (e.g., acetylation or methylation) of the alkaloids (Weid et al., 2004). Likewise, in *Arabidopsis*, the key steps of the glucosinolate biosynthesis are localized in distinct cells, in order to minimize the risk of self-toxication (Fuchs et al., 2016; Nintemann et al., 2018). In Medicago enzymes like PAL and IFS represent enzymatic crossroads in the central phenylpropanoid pathway and isoflavonoid biosynthesis, respectively. Upon biotic stress/elicitation, their activity is crucial for *de novo* production of medicarpin, a major phytoalexin of Medicago (Naoumkina et al., 2007). Among the six *PAL* and three *IFS* genes present in the Medicago genome, those expression of which is highly and peculiarly induced by fungal elicitor, exhibit differential tissue localization in root. The upregulated *PAL* isoforms (*PAL4*, *PAL6*) were mainly expressed in the vascular bundles, while the *IFS*s (*IFS1*, *IFS3*) were generally present in the root cortex (Biala et al., 2017). Spatial separation of enzymatic steps leading to desired product suggests that cooperation between various metabolons in different cells cannot be excluded and sharing of the intermediates in such scenario could also be considered.

## Intracellular Distribution of Phenylpropanoids

**FIGURE 2:**
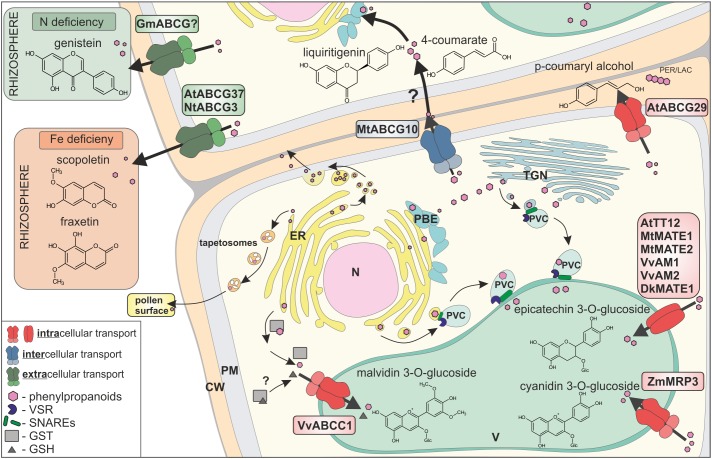
Transport of phenylpropanoids across biological membranes, by three different mechanisms: (i) vesicle trafficking, (ii) gluthatione *S*-transferases (GSTs)-supported, and (iii) membrane transporters. Membrane transporters are distinguished by respective colours, as involved in intracellular transport (red), intercellular transport (blue), and extracellular transport (green). CW, cell wall; PM, plasma membrane; ER, endoplasmic reticulum; N, nucleus; V, vacuole; TGN, *trans*-Golgi network; PVC, pre-vacuolar compartment; PBE, phenylpropanoid biosynthetic enzymes; PER/LAC, peroxidase/laccase; VSR, vacuolar sorting receptor; GST, glutathione *S*-transferase; GSH, glutathione.

In addition to the possibility that certain low-molecular-weight molecules, e.g., 4-coumarate, can disperse by membrane diffusion, three various overlapping, but not exclusive, scenarios of phenylpropanoid distribution exist in planta: (i) vesicle trafficking, (ii) gluthatione *S*-transferases (GSTs)–supported, and (iii) membrane transporter mediated, fulfilled mostly by members of two protein families, namely the MATE (Multidrug and Toxic Compound Extrusion) and ABC (ATP binding cassette) (Figure 2) (Zhao and Dixon, 2010).

All three scenarios are well-described in the intracellular distribution context and have been proposed to collaborate in the circulation of phenylpropanoids (Zhao, 2015). Vesicular transport may proceed in Golgi-dependent or independent manner, and it is controlled by adaptor proteins, like small GTPases and protein complexes such as SNARE (Žárský et al., 2009; Kulich and Žárský, 2014; Singh et al., 2014). It was shown that the distribution of, e.g., anthocyanins, into the vacuole occurs, *inter alia*, by vesicular transport (Winkel-Shirley, 2001; Hsieh and Huang, 2007; Poustka et al., 2007; Gomez et al., 2011; Ichino et al., 2014).

Gluthatione *S*-transferases from various plants, such as petunia (*Petunia hybrida*) Anthocyanin9 (AN9), maize (*Zea mays*) Bronze2 (BZ2), *Arabidopsis* Transparent Testa (TT) 19, and grapevine (*Vitis vinifera)* GST1/GST4 are also essential for anthocyanin and proanthocyanidin vacuolar accumulation (Conn et al., 2008). Two scenarios of GSTs involvement in anthocyanin accumulation were proposed: (i) GST activity and glutathione conjugation, followed by vacuolar sequestration of glutathione-conjugated anthocyanins, as well as (ii) just GST binding to anthocyanin and formation of GST-anthocyanin complexes protecting flavonoids from oxidation and/or guiding them to the central vacuole (Zhao and Dixon, 2010). Interestingly, it was also shown that free glutathione could be required for transport of the anthocyanin, malvidin 3-*O*-glucoside, into the vacuole (Francisco et al., 2013).

Membrane transporters belonging to MATE family from various species like *V. vinifera* (Gomez et al., 2009; Pérez-Díaz et al., 2014), persimmon (*Diospyros kaki*) (Yang et al., 2016), *Arabidopsis* (Marinova et al., 2007), as well as Medicago (Zhao and Dixon, 2009; Zhao et al., 2011) are engaged in the transport of phenylopropanoid glucosides into the vacuole. In Medicago, MATE1 transports epicatechin 3-*O*-glucoside associated with the synthesis of PAs, whereas MATE2 preferentially transports anthocyanin malonates (Zhao and Dixon, 2009; Zhao et al., 2011). Genetic studies have also shown that multidrug resistance-associated proteins (MRP)/C-type of ABC (ABCC) transporters, such as maize MRP3 and grapevine ABCC1, are involved in anthocyanin accumulation, with the assumption that they transport flavonoid conjugates through the tonoplast (Goodman et al., 2004; Francisco et al., 2013).

## Inter- and Extra-Cellular Distribution of Phenylpropanoids

The metabolon formation together with intracellular distribution and, e.g., vacuolar storage, are important for the functioning of phenylpropanoids in particular cells. Less is known about the molecular determinants participating in the intercellular sharing/transport of those molecules. It is worth considering that export to the apoplast could be also a simple way to control, *inter alia*, the concentration of chemically/biologically active intermediates. The latter were shown to negatively modulate the biosynthesis of particular metabolites. For instance, the PAL activity is negatively regulated by *trans*-cinnamic acid and subsequent metabolites (Zhang and Liu, 2015). It was shown that in *Arabidopsis*, the double mutant deficient in UGT78D1 and UGT78D2 (UDP carbohydrate – dependent glycosylotransferases, responsible for flavonol-sugar conjugation) accumulating flavonols, repressed activity of flavonoid-related PAL isoforms as well as CHS, and inhibited flavonol synthesis (Yin et al., 2012). In this respect, the export to the apoplast could be recognized as a regulatory aspect of both the efficiency of cellular biosynthesis and intermediates dispersal.

Two transport mechanisms, (i) vesicular trafficking and (ii) membrane transporters, were shown to mediate phenylpropanoids transport out of the cell. In rape (*Brassica napus*) anthers, the ER derived vesicles – tapetosomes accumulate flavonoids, which are discharged to the pollen surface upon tapetum programmed cell death (Hsieh and Huang, 2007). Phenylpropanoids like, for instance, the hydroxycinnamic acid derivatives, were found in the cell walls, esterified to the wall polysaccharides. Hydroxycinnamic acid derivatives are synthesized at the ER, and from there, they are released as small membrane vesicles, which aggregate into bigger structures, fusing with the plasma membrane and releasing the content into the apoplast (Kulich and Žárský, 2014).

Membrane transporters of the G-type of ABC (ABCG) proteins have been shown to export phenylopropanoids out of the cell. Until recently, it was thought that most of the molecules translocated to the apoplast, by proteins like ABC transporters, are final products of individual biosynthetic pathway, fulfilling a particular biological role. For instance, the flavonoid genistein is suggested to be an exudate from soybean (*Glycine max*) roots, by the G-type of ABC (ABCG) transporters. Genistein, together with daidzein, were found in soybean root exudates, as signaling molecules mediating communication between plant and nitrogen fixing bacteria *Bradyrhizobium japonicum* (Sugiyama et al., 2007, 2008). Scopoletin, belonging to the coumarin subfamily, is synthesized in *Arabidopsis* roots and excreted to the rhizosphere by ABCG37, in order to facilitate Fe nutrition (Fourcroy et al., 2014, 2016; Ziegler et al., 2017). Similarly, in tobacco (*Nicotiana tabacum*), NtPDR3/ABCG3 is involved in secretion of *O*-methylated coumarins, such as fraxetin, to the rhizosphere (Lefèvre et al., 2018). For further reading, see Lefèvre and Boutry (2018).

Interestingly, it was recently shown that ABCG transporters might participate in transporting of the precursors from the phenylpropanoid pathway. The Medicago plasma membrane protein MtABCG10 is responsible for selective translocation of 4-coumarate, an early precursor of the core phenylpropanoid pathway, and liquiritigenin from the 5-deoxyflavonoid branch leading to medicarpin. Upon biotic stress, MtABCG10 action is strictly associated with the 5-deoxyflavonoid branch, even if early precursors like 4-coumarate are common for almost all of the phenylpropanoid products (Banasiak et al., 2013). Biotic stress driven expression of genes encoding enzymes crucial for *de novo* production of medicarpin like *PAL* and *IFS* goes along with *MtABCG10*. Moreover, *PAL* and *MtABCG10* are concomitantly expressed in contrast to *IFS* present in different tissue. Thereby, it was proposed that MtABCG10 appears as a transporter facilitating allocation of common intermediates between various metabolons situated in diverse tissues, upon biotic stress (Biala et al., 2017). Another example is the AtABCG29, which in *Arabidopsis* has been described as a plasma membrane transporter of the *p*-coumaryl alcohol to the cell wall, where this molecule is further oxidized and finally polymerized to lignins (Miao and Liu, 2010; Alejandro et al., 2012). Moreover, it was also proposed that the translocation of the two other lignin precursors, namely sinapyl and coniferyl alcohol, is ABCG dependent (Takeuchi et al., 2018). The expression pattern of those putative transporters was similar to the transcription factor involved in lignin synthesis and lignin polymerization peroxidase (Takeuchi et al., 2018). Interestingly, the dedicated transcriptional network was also determined, underlying the production of benzenoid, as well as phenylpropanoid volatiles, in petunia flowers. This network involves: (i) the ODORANT1 transcription factor which controls biosynthesis in petunia flowers, (ii) a biosynthetic enzyme catalyzing an end-product, and (iii) ABC subfamily G member PhABCG1, a predicted plasma-membrane transporter that is expressed almost exclusively in petals of open flowers. However, the latter transporter is responsible for active transport of emitted volatiles across the plasma membrane, rather than the distribution of intermediates (Adebesin et al., 2017).

## Potential Role of Import

Despite our knowledge on phenylpropanoid biosynthetic enzymes, we are still about to discover how they spatially interact and what are the molecular determinants enabling such interaction. One currently existing gap in the targeted intercellular distribution scenario is the presence/identification of dedicated importers. Indeed, importers are key regulators of intracellular channeling of simple phenolics and/or biosynthesis of different end products. For instance, the full size peroxisomal ABC transporter of *Arabidopsis* ABCD1/PXA1 is indirectly linked to the synthesis of various secondary metabolites such as: (i) benzoic acids, likely by transport of cinnamic acid/cinnamoyl-CoA into the peroxisome (Bussell et al., 2014), (ii) ubiquinone, probably by import of 4-coumarate/coumaroyl-CoA (Block et al., 2014), and (iii) flavonoids by mediation in fatty acids breakdown, which induces flavonoid biosynthetic enzymes (Carrera et al., 2007). From biosynthesis of other secondary metabolites, e.g., root-synthesized glucosinolates in *Arabidopsis*, we have learnt that modulating specific import activities at the plasma membrane level could be essential for shaping the distribution pattern/biosynthesis at the organismal level (Xu et al., 2017). However, our knowledge about import as well as plasma membrane localized importers of phenylpropanoids is still limited.

## Conclusion

Revealing the transport mechanisms/transporters involved in targeted intermediates distribution will bridge the knowledge gaps regarding spatiotemporal phenylpropanoid production under various conditions. It will also facilitate more precise metabolic engineering of those compounds in plants, in order to improve agronomic traits or nutritional value. In the future, metabolons could incorporate switching mechanisms in which metabolic status is sensed, causing association or disassociation of the enzymatic complexes. Such a switching mechanism could rely, *inter alia*, on tightly controlled transport/dedicated transporters, being present in both inter and intracellular scenarios.

## Author Contributions

WB and MJ designed and wrote the manuscript.

## Conflict of Interest Statement

The authors declare that the research was conducted in the absence of any commercial or financial relationships that could be construed as a potential conflict of interest.
